# Association of the HPV vaccination intention‒behavior gap with ehealth literacy and health beliefs among chinese female college students: cross-sectional study

**DOI:** 10.1038/s41598-026-56702-3

**Published:** 2026-06-04

**Authors:** Zheng-Ai Cui, Yuan Li, Yuanju Wang, Min Lu, Xinyue Shi, Ying-Ai Cui

**Affiliations:** 1 Shunde Women and Children’s Hospital of Guangdong Medical University, Foshan, 528300 China; 2 Department of Management and Law, School of Humanities and Management, Guangdong Medical University, Dongguan, 523808 China; 3 Finance Department, Guangdong Medical University, Xincheng Road No. 1, Dongguan, 523808 China; 4 Department of Gynecology, Affiliated Hospital Group of Guangdong Medical University Shenzhen Baoan Central Hospital (Baoan Central Hospital of Shenzhen), Shenzhen, 518100 China; 5 School of Nursing, Guangdong Medical University, Xincheng Road No. 1, Dongguan, 523808 China

**Keywords:** HPV vaccine, eHealth literacy, Intention‒behavior gap, Health belief model, Female college students, Trust, Diseases, Health care, Psychology, Psychology

## Abstract

With the strengthening of public health, Chinese female college students have shown higher levels of HPV vaccination intention, but the actual vaccination rate is very low. This significant intention–behavior gap has become a major challenge for the prevention and control of HPV among colleges and universities. This study aims to explore the intention–behavior gap for HPV vaccine uptake among Chinese female college students on the basis of eHealth literacy (eHL) and the health belief model (HBM). A cross-sectional survey was conducted from June 16 to July 16, 2024, to assess socioeconomic status, eHL and HBM among female college students at Guangdong Medical University. Descriptive statistics were calculated, and binary logistic regression analysis was performed using SPSS 29.0 to identify significant independent associations between female college students’ HPV vaccination intentions and behavior. Among the 2884 valid participants with an intention to receive an HPV vaccination, only 41.3% had converted from intention to uptake (≥ 1 dose).The key influencing factors included being from urban areas (adjusted OR: 1.24; 95% CI 1.04– 1.47; *p* = 0.018), monthly consumption (adjusted OR: 1.24; 95% CI 1.13–1.37; *p* < 0.001), household income (adjusted OR: 1.10; 95% CI 1.01 –1.21; *p* = 0.029), and parental education level (adjusted OR: 1.23; 95% CI 1.13–1.33; *p* < 0.001). Moreover, the participants’ eHL level positively influenced their HPV vaccination conversion rate (adjusted OR: 1.02; 95% CI 1.00–1.04; *p* = 0.036). For HBM, trust in formal information had a significant positive influence on HPV vaccination conversion rate (adjusted OR: 1.10; 95% CI 1.04–1.16; *p* = 0.002), whereas perceived behavioral barriers had a significant negative influence (adjusted OR: 0.72; 95% CI 0.66 – 0.79; *p* < 0.001). This study confirms a substantial HPV vaccination intention‒behavior gap among Chinese female college students, influenced by socioeconomic factors, eHL, and health beliefs—specifically trust in formal information and perceived barriers. Therefore, we suggest that targeted measures should be undertaken to reduce socioeconomic gaps, improve the eHL of those with pre-existing vaccination intention, enhance institutional trust in formal medical institutions and authoritative information channels, and reduce the psychological and practical obstacles to HPV vaccination to effectively bridge the intention–behavior gap and thereby improve vaccination coverage female college students in China.

## Introduction

Human papillomavirus (HPV) vaccination is globally recognized as the most cost-effective strategy for preventing cervical cancer. Globally, HPV vaccination coverage remains suboptimal despite the proven efficacy of the vaccine. According to the World Health Organization (WHO), global coverage of the first dose of HPV vaccine among girls aged 9–14 years old increased from 17% in 2019 to 31% in 2024, still far below the 90% target for cervical cancer elimination by 2030^1^. Coverage varies substantially across regions: high-income countries achieved a first-dose coverage of 56% among adolescent girls in 2023, compared with only 23% in low- and middle-income countries^2^. Even in high-income settings, a notable discrepancy between vaccination intention and actual uptake has been documented. Studies conducted in the United States have shown that while HPV vaccination intent among college students can be high, actual series completion rates remain low^3^. In Europe, coverage rates vary widely—only two countries achieved 90% uptake of girls receiving all vaccine doses, while several countries had rates below 50%^4^. This intention–behavior gap is therefore not unique to any single healthcare system but represents a broader public health challenge in the promotion of HPV immunization.

However, the vaccination rate of Chinese female college students, as the key supplementary vaccination population for the vaccine, is still not optimistic. The latest comprehensive report of the Chinese Center for Disease Control and Prevention revealed that in 2022, the coverage rate of the first dose of the HPV vaccine for people aged 20–24 years was the highest, but only 14.02%^5^, and the overall coverage rate remained far below the ≥ 50% coverage threshold associated with substantial herd protection^6^, and well below the WHO’s 90% target for cervical cancer elimination by 2030 ^7^. The literature lacks comprehensive data on HPV vaccination rates among female college students in China, but limited survey results have demonstrated low vaccination rates^8–10^. Despite the popularization of public health education, this group has shown high levels of vaccination intention^11^; however, this high intention has not been effectively transformed into actual vaccination behavior. This significant “intention–behavior gap” has become the main challenge for HPV prevention and control in colleges and universities^12^. Given that college is an early stage of individual sexual activity and represents a critical period for preventing HPV transmission through immunization^7^, an in-depth understanding of and addressing this “intention–behavior gap” are essential for reducing the burden of HPV-related diseases such as cervical cancer in the future.

Numerous previous studies have applied the Health Belief Model (HBM) extensively to predict individuals’ vaccination decisions and have confirmed that perceived susceptibility, perceived severity, and perceived barriers are key predictors of vaccination willingness^9,13^. However, there are obvious limitations in the existing research. First, in most studies, “vaccination intention” is treated solely as an endpoint indicator, assuming that intention directly predicts behavior without thoroughly analyzing the gap between intention and action. This approach fails to adequately explain why many students with positive intentions ultimately do not complete vaccination^14^. Second, in the digital information age, the internet has become the primary channel through which college students access health information, making eHealth literacy (eHL) increasingly crucial. As the core competency for individuals to acquire, comprehend, and evaluate online health information, it is considered to be a significant factor influencing health decisions^15^. Studies have shown that eHL is positively associated with HPV vaccination intention or uptake among college students^16–18^. However, its specific role in the transition from the intention to receive an HPV vaccination to actual behavior has not been systematically elucidated. There remains a lack of empirical research that integrates it with the HBM and puts them together under the “intention–behavior gap” explanatory framework, especially in the context of Chinese social culture.

In view of the above research deficiencies, it is necessary to systematically explore the gap between HPV vaccination intention and behavior among Chinese female college students and analyze the role of eHL and health beliefs in this gap. The present study employs a cross-sectional survey design, which targets female college students at Guangdong Medical University. Data on their HPV vaccination intention, vaccination behavior, eHL, and health belief-related indicators were collected to analyze the occurrence of the intention–behavior gap and its influencing factors. The objectives of this study were (1) to quantify the magnitude of the gap between vaccination intention and actual behavior within this population and (2) to examine the independent association of eHL and dimensions of health beliefs with the gap between HPV vaccination intention and behavior. Our findings provide a theoretical foundation and empirical support for the development of targeted HPV vaccine promotion and health education strategies in higher education settings.

## Materials and methods

### Survey design

This study was conducted at Guangdong Medical University from June 16 to July 16, 2024. The university was selected due to its large, geographically diverse female student body and the research team’s institutional access, which enabled efficient data collection and ensured broad representation of socioeconomic backgrounds within the region. Using a convenience sampling method, an online questionnaire survey was administered through the Chinese online survey platform “Wenjuanxing” (www.wjx.cn). This approach was adopted because a complete and accessible sampling frame of all full-time students was unavailable to the research team, and the one-month data collection window precluded the administrative coordination necessary for probability sampling.

### Study participants

The survey was distributed to full-time students at the university via WeChat. The sample size was calculated a priori using the following formula on the basis of an error α of 0.05 and a permissible error δ of 0.05:

$$n=\frac{{Z}_{1-\alpha\:/2}^{2}\ast\:p(1-p)}{{\delta\:}^{2}}$$

where *n* represents the sample size required for the survey, ${Z}_{1-\alpha\:/2}^{2}$ is the standard normal deviation of α, and *p* denotes the expected prevalence or positive rate of the survey, specifically referring to the HPV vaccination rates among Chinese college students. In this study, the proportion *p* was conservatively set at 0.5 to ensure the calculation of the largest minimum sample size. To account for the potential impact of invalid questionnaires, the sample size for this study was conservatively increased by 20%. Consequently, the final minimum sample size was 461 male and 461 female college students, resulting in a total minimum sample size of 922 participants.

The inclusion criteria were as follows: (1) full-time enrolled students; (2) completion and successful submission of all mandatory questionnaire items, excluding questions skipped because of branching logic. The exclusion criteria were as follows: (1) a response time of less than 180 s; (2) inconsistencies and illogical responses in some answers; (3) failure to provide accurate academic and professional information.

**Fig. 1:**
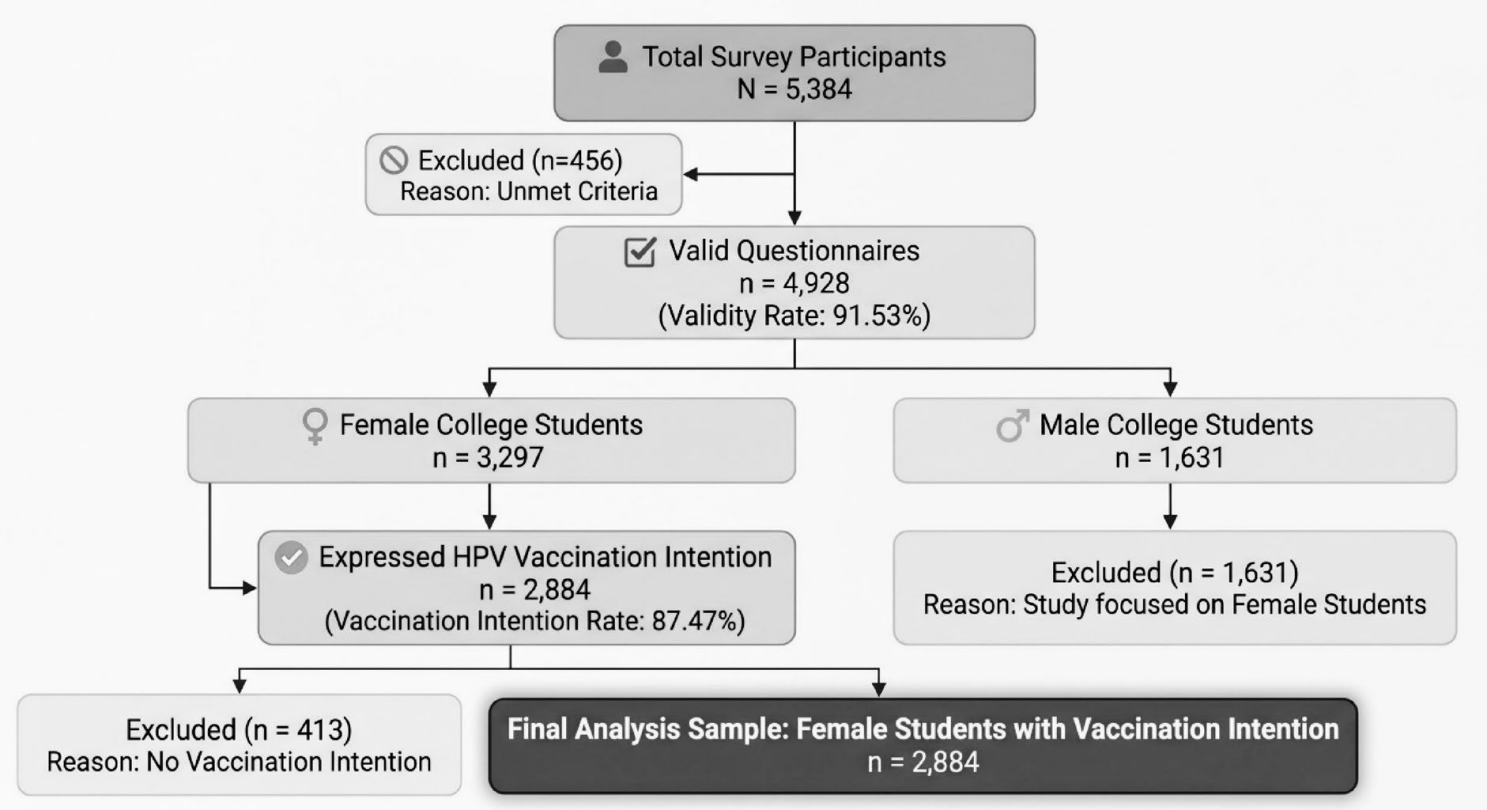
Participant flow and selection process.

A total of 5384 individuals participated in the survey, with 456 responses excluded because of unmet criteria. This yielded 4928 valid questionnaires (validity rate: 91.53%). Among the valid responses, 3297 participants were female college students, and 1631 were male college students. Male respondents were not included in the present analysis, as the study objective focused specifically on HPV vaccination intention and behavior among female college students. Among the female college students, 2884 participants (vaccination intention rate: 87.47%) expressed an intention to receive the HPV vaccine. Therefore, the final analysis included 2884 female college students who had expressed vaccination intention; female students without such intention were excluded from subsequent analyses (Fig. 1). It should be noted that the study findings are specific to female students with a pre-existing intention to vaccinate and do not necessarily generalize to the broader population of female college students without such intention.

### Ethical considerations

This study was approved by the Clinical Research Ethics Committee of the Affiliated Hospital of Guangdong Medical University (approval NO. PJKT2023-126). The study participants read and signed an online informed consent form. Potential study participants were informed that participation in this study was voluntary and that they could withdraw their decision to participate at any time.

### Variables and measurements

The survey included (1) socioeconomic and demographic characteristics, (2) the eHL scale, and (3) the health belief model.

### Sociodemographic characteristics

The following characteristics were assessed in this survey: household registration, monthly consumption, highest educational background of parents, monthly household income, and HPV vaccination status (Table 1). Given the ordered nature of monthly consumption, highest educational background of parents, monthly household income, these variables were recoded into ordinal scores at the individual level for non-parametric analysis. Specifically, monthly consumption was coded as 1 = < 1000 CNY, 2 = 1000–1500 CNY, 3 = 1501–2000 CNY, 4 = 2001–3000 CNY and 5 = > 3000 CNY. Highest educational background of parents were coded as 1 = Junior high school or below, 2 = High school or technical secondary school, 3 = Associate’s degree, 4 = Bachelor’s degree, and 5 = Master’s degree or above. Monthly household income was coded as 1 = < 1500 CNY, 2 = 1500–2500 CNY, 3 = 2501–4000 CNY, 4 = 4001–8000 CNY, and 5 = > 8000 CNY. These ordinal scores were used in subsequent analyses and were treated as continuous variables reflecting increasing levels.

**Table 1:** Sociodemographic characteristics of the participants.

Variable	Total, *N* (%)
*Sociodemographic characteristics*	
Household Registration	
Rural	1546 (53.6)
Cities and Towns	1338 (46.4)
Monthly Consumption (CNY)*	
< 1000 ($140.65)	223 (7.7)
1000–1500 ($140.65-$210.97)	1377 (47.8)
1501–2000 ($211.11-$281.29)	901 (31.2)
2001–3000 ($281.43-$421.94)	300 (10.4)
> 3000 ($421.94)	83 (2.9)
The Highest Educational Background of Parents	
Junior high school or below	1103 (38.3)
High school or technical secondary school	956 (33.2)
Associate’s degree	393 (13.6)
Bachelor’s degree	385 (13.4)
Master’s degree or above	47 (1.6)
Monthly household income (CNY)*	
< 1500 ($210.97)	72 (2.5)
1500–2500 ($210.97-$351.62)	177 (6.1)
2501–4000 ($351.76-$562.59)	455 (15.8)
4001–8000 ($562.73-$1125.18)	982 (34.1)
> 8000 ($1125.18)	1198 (41.5)
HPV vaccination status	
No	1694 (58.7)
Yes	1190 (41.3)

*Exchange rate on June 16, 2024: 1 USD = 7.12 CNY, same below.

The intention‒behavior gap for HPV vaccination denoted the nonconversion of intention into action. In this study, it was assessed by examining the actual vaccination status of those participants with vaccination intention. Actual vaccination was defined as having received at least one dose of the HPV vaccine, as ascertained by self-report. This definition was adopted for two primary reasons. First, given the cross-sectional design, this study focused on the critical behavioral transition from intention to initiation, which represents a pivotal step in the vaccination decision-making process. Second, series completion requires a longer observation window, and the cross-sectional design cannot distinguish between those who have not yet completed the series due to time constraints from those who have truly abandoned vaccination. Using ≥ 1 dose thus captures the actual behavioral response to intention at the time of survey, allowing for a more focused investigation of the intention–behavior gap as influenced by eHL and health beliefs.

### eHealth literacy scale

The Chinese adaptation of the 8-item eHL scale originally validated by Dong and colleagues served as the study measurement tool^19^ (Table 2). The authors obtained permission to use this instrument from the copyright holders. A 5-point Likert-type scoring system was used (1 = strongly disagree to 5 = strongly agree). The total eHL score was calculated by summing the responses to all 8 items, yielding a possible score range of 8–40, with higher scores indicating higher eHL competencies. Internal consistency analysis revealed excellent scale reliability (Cronbach’s α = 0.938).

**Table 2:** Descriptive statistics of eHealth literacy.

Term	Strongly disagree *N* (%)	Disagree *N* (%)	Neutral/Unsure *N* (%)	Agree *N* (%)	Strongly agree *N* (%)	Score (M ± SD)
1. I know what health resources are available on the internet	19 (0.7)	51 (1.8)	423 (14.7)	1775 (61.5)	616 (21.4)	4.01 ± 0.70
2. I know where to find helpful health resources on the internet	19 (0.7)	76 (2.6)	540 (18.7)	1670 (57.9)	579 (20.1)	3.94 ± 0.74
3. I know how to find helpful health resources on the internet	19 (0.7)	78 (2.7)	504 (17.5)	1696 (58.8)	587 (20.4)	3.96 ± 0.74
4. I know how to use the internet to answer my health questions	17 (0.6)	65 (2.3)	424 (14.7)	1766 (61.2)	612 (21.2)	4.00 ± 0.71
5. I know how to use the health information I find on the internet to help me	12 (0.4)	37 (1.3)	267 (9.3)	1919 (66.5)	649 (22.5)	4.09 ± 0.63
6. I have the skills I need to evaluate the health resources I find on the internet	16 (0.6)	75 (2.6)	538 (18.7)	1688 (58.5)	567 (19.7)	3.94 ± 0.73
7. I can tell high-quality from low-quality health resources on the internet	9 (0.3)	74 (2.6)	539 (18.7)	1694 (58.7)	568 (19.7)	3.95 ± 0.72
8. I feel confident in using information from the internet to make health decisions	20 (0.7)	85 (2.9)	438 (15.2)	1753 (60.8)	588 (20.4)	3.97 ± 0.73
eHealth Literacy						31.87 ± 4.76

### Health beliefs

In this study, we employed the health belief model to measure participants’ health beliefs regarding HPV vaccination. The HBM utilized in this study was validated by Chen et al.^20^ (Table 3). This scale includes 13 items across five dimensions: perceived benefits of behavior (3 items; score range: 3–15), trust in formal information (4 items; score range: 4–20), perceived disease severity (2 items; score range: 2–10), perceived disease susceptibility (3 items; score range: 3–15), and perceived behavioral barriers (1 item; score range: 1–5). Each item was also scored on a 5-point Likert scale. For each dimension, the score was calculated by summing the responses to its constituent items. No total HBM score was computed, as the dimensions represent conceptually distinct constructs with different directional meanings. Each dimension was analyzed separately in subsequent analyses. The Cronbach’s α value for the scale was 0.719.

**Table 3:** Descriptive statistics of HBM.

Term	Strongly disagree *N* (%)	Disagree *N* (%)	Neutral/Unsure *N* (%)	Agree *N* (%)	Strongly agree *N* (%)	Score (M ± SD)
1. I think HPV vaccines are important for protecting myself from HPV infection and related diseases	5 (0.2)	13 (0.5)	103 (3.6)	1639 (56.8)	1124 (39.0)	4.32 ± 0.65
2. I think the safety of the HPV vaccine can be guaranteed	6 (0.2)	28 (1.0)	448 (15.5)	1659 (57.5)	743 (25.8)	4.18 ± 0.66
3. I think the HPV vaccine is effective	7 (0.2)	8 (0.3)	240 (8.3)	1779 (61.7)	850 (29.5)	4.21 ± 0.67
*Perceived benefits of behavior*						12.71 ± 1.78
4. I trust the HPV vaccine knowledge and services provided by the vaccination clinic and doctor	8 (0.3)	10 (0.3)	266 (9.2)	1741 (60.4)	859 (29.8)	4.22 ± 0.63
5. I trust the HPV vaccine knowledge and services provided by CDC, hospitals and other professional institutions	6 (0.2)	8 (0.3)	196 (6.8)	1762 (61.1)	912 (31.6)	4.28 ± 0.62
6. I trust HPV vaccine manufacturers and companies	7 (0.2)	53 (1.8)	748 (25.9)	1435 (49.8)	641 (22.2)	3.96 ± 0.84
7. I trust the HPV vaccine information provided by the government	5 (0.2)	7 (0.2)	179 (6.2)	1765 (61.2)	928 (32.2)	4.29 ± 0.57
*Trust in formal information*						16.75 ± 2.42
8. HPV infection may have serious adverse effects on my health	77 (2.7)	257 (8.9)	420 (14.6)	1136 (39.4)	994 (34.5)	3.99 ± 1.09
9. I think cervical cancer is a serious disease	18 (0.6)	78 (2.7)	199 (6.9)	1342 (46.5)	1247 (43.2)	4.39 ± 0.74
*Perceived disease severity*						8.38 ± 1.51
10. I don’t think HPV infection can be avoided	571 (19.8)	1453 (50.4)	572 (19.8)	231 (8.0)	57 (2.0)	2.25 ± 1.00
11. I think infection by HPV is because of sexual arbitrariness	427 (14.8)	940 (32.6)	624 (21.6)	757 (26.2)	136 (4.7)	2.67 ± 1.09
12. I’m in good health and I’m not afraid of getting HPV	1315 (45.6)	1197 (41.5)	206 (7.1)	113 (3.9)	53 (1.8)	1.69 ± 0.92
*Perceived disease susceptibility*						6.61 ± 2.32
13. I am very cautious about “getting the HPV vaccine”	62 (2.1)	307 (10.6)	488 (16.9)	1566 (54.3)	461 (16.0)	3.73 ± 0.93
*Perceived behavioral barriers*						3.73 ± 0.93

Each dimension score of HBM was calculated as the sum of its constituent items.

### Data analysis methods

Data analysis was performed using SPSS 29.0 (IBM Corporation, Armonk, NY, USA). Continuous variables are summarized as the mean (M) ± standard deviation (SD), whereas categorical variables are reported as counts (N) and proportions (%). The normality of continuous variables was assessed using the Kolmogorov–Smirnov test and Q–Q plots. As most continuous variables did not follow a normal distribution, nonparametric tests were applied for group comparisons. The Mann–Whitney U test was used for comparisons between two independent groups, while the Kruskal–Wallis test was employed for comparisons among three or more independent groups. For the ordered categorical variables (monthly consumption, parental education, and household income), the ordinal scores assigned as described in the Variables and measurements section were used. The Kruskal–Wallis test was applied to compare the distributions of these ordinal scores across the HPV vaccination intention–behavior gap groups, and the results were presented as median and interquartile range (IQR). As participants were recruited through convenience sampling without cluster-based enrollment (e.g., by class or dormitory) and responded independently via an online questionnaire, we assumed independence of observations for the purpose of statistical testing. Univariate analyses were conducted to examine differences in demographics, eHL, and various dimensions of the HBM between groups defined by vaccination status (i.e., intention converted to vaccination vs. intention not converted). For the binary logistic regression analysis, ordered categorical variables (monthly consumption, parental education, and household income) were entered as continuous ordinal scores to estimate the linear trend in odds for each one-level increase. Variables with *p* < 0.05 in the univariate analyses were entered into a multivariable binary logistic regression model to identify significant independent associations with HPV vaccination intention–behavior gap among college students. Adjusted odds ratios (ORs) with 95% confidence intervals (CIs) were computed, with statistical significance set at *p* < 0.05.

## Results

### Sociodemographic characteristics

Among the 2,884 valid participants, 53.6% were from rural areas, and 79.0% reported monthly personal expenditures between 1,000 and 2,000 CNY. Parental education was relatively low, with 71.4% having a high school diploma or less. In contrast, 75.6% of the households had a monthly income exceeding 4,000 CNY.

Most significantly, an intention–behavior gap was observed for HPV vaccination: among these students with vaccination intention, only 41.3% subsequently received the vaccine, with 58.7% failing to translate their willingness into actual vaccination (Table 1).

### eHealth literacy

The mean total eHL score of the female college students in the sample was 31.87 ± 4.76. This total corresponded to a per-item average of 3.98, which aligned with established thresholds for advanced eHL (Table 2).

### Health beliefs

The scores for the HBM dimensions were as follows: perceived benefits of behavior (12.71 ± 1.78), trust in formal information (16.75 ± 2.42), perceived disease severity (8.38 ± 1.51), perceived disease susceptibility (6.61 ± 2.32), and perceived behavioral barriers (3.73 ± 0.93) (Table 3).

### Univariate analyses

Univariate analysis revealed significant differences between HPV vaccination intention–behavior gap and key variables, as detailed in Table 4. Household registration, monthly consumption, highest educational background of parents, and monthly household income (all *p* < 0.001) demonstrated particularly significant differences.

**Table 4:** Univariate differences by HPV vaccination intention–behavior gap.

Variable	HPV vaccination status *N* (%)		*p*
	No	Yes	
*Sociodemographic characteristics*			
Household Registration			**< 0.001** ^**a**^
Rural	1011 (59.7)	535 (45.0)	
Cities and Towns	683 (40.3)	655 (55.0)	
Monthly Consumption, median (IQR)	2.0(2.0,3.0)	3.0(2.0,3.0)	**< 0.001** ^**c**^
The Highest Educational Background of Parents, median (IQR)	2.0(1.0,2.0)	2.0(1.0,3.0)	**< 0.001** ^**c**^
Monthly Household Income, median (IQR)	4.0(3.0,5.0)	5.0(4.0,5.0)	**< 0.001** ^**c**^
eHealth Literacy (M ± SD)	31.50 ± 4.80	32.40 ± 4.60	**< 0.001** ^**b**^
HBM (M ± SD)			
Perceived Benefits of Behavior	12.55 ± 1.96	12.98 ± 1.44	**< 0.001** ^**b**^
Trust in Formal Information	16.55 ± 2.38	17.08 ± 2.49	**< 0.001** ^**b**^
Perceived Disease Severity	8.27 ± 1.33	8.55 ± 1.77	**0.034**
Perceived Disease Susceptibility	6.73 ± 2.32	6.42 ± 2.33	**< 0.001** ^**b**^
Perceived behavioral barriers	3.79 ± 0.85	3.63 ± 1.05	**< 0.001** ^**b**^

a: *p* value from the Pearson chi-square test. b: *p* value from the Mann‒Whitney test. c: values are presented as median (IQR) of the ordinal scores. Group differences were tested using the Kruskal‒Wallis test. Boldface indicates statistical significance (*p* < 0.05).

Notably, the difference in the intention–behavior gap regarding HPV vaccination among groups with the eHL level and various dimensions of the HBM were statistically significant (all *p* < 0.05).

### Factors associated with the HPV vaccination intention–ehavior gap

The variables that were found to be significant in the univariate analysis were subsequently included in a binary logistic regression analysis. The results are presented in Table 5.

**Table 5:** Binary logistic regression for the HPV vaccination intention–behavior gap.

Variable	Logit coefficient	Adjusted OR (95% CI)	*p*
Sociodemographic characteristics			
Household Registration	0.212	1.24 (1.04–1.47)	**0.018**
Monthly Consumption	0.217	1.24 (1.13–1.37)	**< 0.001**
The Highest Educational Background of Parents	0.205	1.23 (1.13–1.33)	**< 0.001**
Monthly Household Income	0.098	1.10 (1.01–1.21)	**0.029**
eHealth Literacy	0.020	1.02 (1.00–1.04)	**0.036**
HBM			
Perceived Benefits of Behavior	− 0.008	0.99 (0.92–1.08)	0.839
Trust in Formal Information	0.093	1.10 (1.04–1.16)	**0.002**
Perceived Disease Severity	− 0.021	0.98 (0.93–1.04)	0.466
Perceived Disease Susceptibility	− 0.012	0.99 (0.95–1.02)	0.494
Perceived behavioral barriers	− 0.323	0.72 (0.66–0.79)	**< 0.001**

OR: odds ratio; CI: confidence interval.

Reference categories: Household Registration = Rural; Monthly Consumption = < 1,000 CNY; The Highest Educational Background of Parents = Junior high school or below; Monthly Household Income = < 1500 CNY. Ordered categorical variables (monthly consumption, parental education, and household income) were entered as ordinal scores in the logistic regression models to assess linear trend; thus a single adjusted OR is reported for each one-level increase.

Boldface indicates statistical significance (*p* < 0.05). Hosmer–Lemeshow test, χ^2^(8) = 4.805, *p* = 0.778.

The following socioeconomic and demographic factors were found to have significant associations in the HPV vaccination intention–behavior gap: household registration, monthly consumption, the highest educational background of parents, and monthly household income.

Compared with rural students, students from urban areas presented a significantly greater HPV vaccination rate (adjusted OR: 1.24; 95% CI 1.04–1.47; *p* = 0.018). That is, there was a smaller HPV vaccination intention–behavior gap and a higher conversion rate from intention to action (same below). The average monthly consumption level, average monthly household income and parental education level had a significant positive influence on HPV vaccination (adjusted OR: 1.24; 95% CI 1.13–1.37; *p* < 0.001, adjusted OR: 1.10; 95% CI 1.01–1.21; *p* = 0.029 and adjusted OR: 1.23; 95% CI 1.13–1.33; *p* < 0.001 , respectively).

Moreover, the participants’ eHL positively influenced their conversion rates from intention to uptake (adjusted OR: 1.02; 95% CI 1.00–1.04; *p* = 0.036).

Furthermore, for HBM, trust in formal information had a significant positive influence on the conversion rate from intention to uptake (adjusted OR: 1.10; 95% CI 1.04–1.16; *p* = 0.002), whereas perceived behavioral barriers had a significant negative influence on the conversion rate (adjusted OR: 0.72; 95% CI 0.66–0.79; *p* < 0.001). The other dimensions of the HBM were not significantly associated with the conversion rate.

## Discussion

This study revealed a significant intention–behavior gap regarding HPV vaccination among Chinese female college students, with fewer than half of those pre-existing intention actually receiving the vaccine. High socioeconomic status (e.g., urban household registration and income) and eHL promoted the conversion of vaccination behavior. With respect to health beliefs, trust in formal information acted as a positive factor, whereas perceived behavioral barriers served as a negative factor. These findings suggested that interventions should address socioeconomic factors, individual eHL, and health beliefs to narrow the HPV vaccine intention–behavior gap and increase vaccination rates.

### HPV vaccination intention–behavior gap

In this study, we reported a significant gap between HPV vaccination intentions and actual behavior among Chinese female college students, a phenomenon observed both domestically and internationally^21,22^. Although some countries have incorporated HPV vaccines into their national immunization programs, this gap is particularly pronounced in low- and middle-income countries^23^. National programs that offer free or subsidized vaccines and deliver them through school-based or primary care settings have been shown to substantially increase uptake and reduce intention–behavior discrepancies^23,24^. In contrast, in settings where vaccination relies heavily on individual out-of-pocket payment and opportunistic health-seeking, the translation of intention into action remains suboptimal^25^. This discrepancy indicates that the high willingness rate does not always translate into the vaccination behavior of the population, which may be explained by the existence of non-attitudinal barriers identified in the survey. High willingness may be constrained by practical factors such as socioeconomic status and vaccine cost, as well as beliefs regarding vaccine safety and efficacy, contributing to the observed behavioral disconnection^21,26^. These findings underscore the importance of considering practical and context-specific factors when designing interventions to increase HPV vaccination rates.

### Sociodemographic characteristics

The present study revealed that urban–rural background, household income, monthly consumption level, and parental education served as significant factors influencing the intention–behavior gap in HPV vaccination among Chinese female college students. Collectively, these factors point to the core mechanism of “social determinants of health”—that is, individuals’ socioeconomic status and structural resource accessibility (such as health care coverage, information access, and affordability thresholds) systematically constrain the transition from health intentions to actual behavior^27–30^. These findings align strongly with the literature on vaccination inequities, which collectively underscore the critical role of economic barriers and the unequal distribution of cognitive resources in health behaviors^31–34^. This study further verified the universality of this mechanism among a specific population of Chinese female college students who already had vaccination intention. On this basis, public health policies that focus solely on increasing vaccination willingness while neglecting the removal of structural barriers will yield limited effects. Therefore, we recommend that interventions need to be designed with precise socioeconomic stratification, for example, by providing targeted subsidies and nearby vaccination services for students from rural and low-income families^35^ and conducting accessible vaccine knowledge dissemination for low-education parents^36^. Such an approach will reduce socioeconomic disparities and improve health care accessibility for vulnerable populations and truly bridge the final gap between willingness and action.

### eHealth literacy

The results showed a significant positive correlation between the conversion of HPV vaccination behavior and eHL level among Chinese female college students. Specifically, the greater the eHL, the smaller was the gap between vaccination intention and actual behavior. This association is likely explained by the capacity of higher eHL to enable individuals to more effectively access, understand, and apply online health information, thereby facilitating the translation of vaccination intention into actual action^37^. These findings align with those of previous research concluding that eHL can promote the realization of healthy behavior^38^ but highlight the unique role of eHL in bridging the “intention–behavior gap” within the context of digital health. Therefore, it is necessary to design targeted health education and digital communication strategies to enhance female college students’ eHL. Strengthening their information access and application capabilities can help reduce the “knowledge–behavior gap” phenomenon in the context of HPV vaccination.

### Health beliefs

This study confirmed that the actual conversion of HPV vaccination behavior among female college students in China was significantly positively correlated with their level of trust in formal information sources such as vaccination clinics and doctors, disease control centers and hospitals, and vaccine manufacturers. These findings suggest that, in the context of voluntary vaccination, high levels of institutional and professional trust serve as key risk-reduction mechanisms. Such trust may help mitigate uncertainty in individual vaccination decision-making processes, effectively bridging the gap between health awareness and behavioral implementation. In contrast, the subsequent impact of distrust may stem from certain health beliefs about vaccine efficacy and safety, which can lead to poor HPV vaccination uptake and completion rates^39^. The suspension of HPV vaccination in Japan for seven years is a classic cautionary tale^40^. The present findings are broadly consistent with those of previous studies^41^. A multinational survey revealed that trust in the government, especially in medical institutions, is significantly positively correlated with vaccination^42^. Trust is a key determinant of vaccine hesitancy, and pro-vaccine advocacy campaigns can successfully target high-risk hesitant groups^43^. However, this study further focuses on female college students who already had vaccination intention and supplements empirical evidence on the relationship between trust in formal information and HPV vaccination in the Chinese context. Therefore, for those with pre-existing intention among Chinese female college students, the public health strategy to improve the HPV vaccination rate should shift from simple knowledge popularization to prioritizing the enhancement of institutional trust in formal medical institutions and authoritative information channels. By systematically improving the transparency and credibility of the health care system, these strategies can effectively guide vaccination decisions.

The present study also revealed that the HPV vaccination conversion rate among Chinese female college students was significantly negatively correlated with perceived barriers to vaccination. Compared with perceived severity or susceptibility, perceived barriers emerged as a more potent factor influencing vaccination decisions. These findings suggest that although the target population may be aware of the risk of HPV-related diseases such as cervical cancer, substantial barriers—such as concerns about the cost, safety, and availability of HPV vaccines—serve as significant deterrents and inhibit the conversion of vaccination willingness to actual behavior^44^. This negative correlation confirms the prior application of HBM, which is consistent with the findings of earlier studies^45–47^, indicating that perceived barriers are the core psychological determinants of vaccination decision-making, not just objective constraints. Therefore, in vaccination promotion efforts targeting female college students who already had vaccination intention in China, in addition to disseminating knowledge, priority should be given to implementing targeted measures such as providing clear economic subsidy information, establishing convenient vaccination channels, and sharing empirical evidence concerning safety. Such approaches directly reduce psychological and practical barriers, thereby effectively increasing vaccination coverage.

In the adjusted model, perceived disease susceptibility, perceived disease severity, and perceived benefits of behavior were not significantly associated with the intention–behavior gap. Although central to the HBM, their null associations may reflect several factors. First, Chinese female college students are relatively young and may perceive HPV infection as a distant health threat with low immediate risk^48^. Second, this population—particularly medical students—exhibits generally high HPV awareness, potentially producing a ceiling effect that limits discriminative power^49^. Third, once vaccination intention is formed, actual or perceived barriers (e.g., cost, accessibility, time constraints) and enabling factors (e.g., high eHL in this sample) may outweigh the influence of threat- and benefit-related beliefs^8^. These findings suggest that, in this population, trust in formal information and perceived barriers play a more central role in the intention–behavior gap than do risk perception or benefit beliefs. Interventions that address structural and practical obstacles may therefore be more effective than those targeting threat appraisal alone.

### Limitations

This study has several limitations. First, as a cross-sectional survey, it cannot establish causal relationships between variables. Second, although known confounding factors were controlled for through multivariable analysis, unmeasured confounding variables may still exist. Third, the data relied on self-reports, which are potentially subject to social desirability bias and recall bias. In particular, actual vaccination was assessed via lifetime history without a specified recall period, which may exacerbate recall inaccuracy and precludes determination of the temporal sequence between intention formation and vaccine uptake. Fourth, the sample was obtained through convenience sampling, although the sample size was large, its representativeness may be limited, and conclusions should be extrapolated with caution. Additionally, statistical analyses assumed independence of observations; however, potential peer-network effects among college students may have introduced mild clustering that could not be assessed due to the absence of cluster-level identifiers. Although the participating university enrolls students from diverse geographic and socioeconomic backgrounds, the non-probability nature of recruitment introduces potential selection bias. Future studies employing multi-stage probability sampling across multiple institutions are needed to confirm our results. Fifth, our analysis was restricted to female students who reported intention to receive HPV vaccination; therefore, the observed associations may not apply to those who have no intention to vaccinate. Sixth, monthly consumption, parental education, and household income were modelled as ordinal scores assuming a logit-linear relationship with the intention–behavior gap. This assumption may not fully hold across all variable levels, though it was adopted to preserve model parsimony and statistical power. And, future research should focus on longitudinal studies and interventions to improve the current situation of insufficient HPV vaccination coverage.

## Conclusions

This study assessed the intention–behavior gap among Chinese female college students in Dongguan, China. The findings revealed a persistent significant gap in intention–behavior among Chinese female college students. This gap was affected by “social determinants of health”, such as urban and rural background, household income, monthly consumption level and parental education. In addition, eHL had a positive association with the conversion behavior of HPV vaccination, and dimensions of the HBM—trust in formal information and perceived barriers to vaccination—influenced the transformation of vaccination intention to actual behavior.

Therefore, we recommend implementing precise socioeconomic stratification to address socioeconomic disparities and designing targeted health education and digital communication strategies to enhance female college students’ eHL. Public health strategies to improve the HPV vaccination rate should shift from mere knowledge dissemination to prioritizing the enhancement of institutional trust in formal medical institutions and authoritative information channels. Targeted measures such as providing clear economic subsidy information, establishing convenient vaccination channels, and sharing evidence of safety should be prioritized to directly reduce psychological and practical barriers, thereby effectively increasing HPV vaccination coverage among female college students who already had vaccination intention.

## Data Availability

The datasets generated during and/or analyzed during the current study are not publicly available because of personal privacy or moral and ethical issues but are available from the corresponding author upon reasonable request.
